# Supplementary material for: Redesigning metabolism based on orthogonality principles
Source: Nat Commun. 2017 May 30;8:15188. doi: 10.1038/ncomms15188 (PMC5459945; doi:10.1038/ncomms15188)
Supplement: Supplementary Information — Supplementary figures, supplementary tables, supplementary notes, supplementary methods and supplementary references. [file ncomms15188-s1.docx]

**Supplementary Note 1**

**Synthetic pathway design**

Pathway design requires consideration for three specific aspects of any metabolic pathway; the desired product to be produced, the input substrate and the kinetics of enzyme associated with the pathway.  In addition, they also need to consider network energy and redox constraints. We cover these criteria in detail below.

One important consideration is that the pathways we model are a simplification of the cell’s metabolism and we purposefully at this stage neglect the complexity that arises by considering the metabolism in more detail.  For example, we largely neglect amino acid biosynthesis pathways in the cell.  We do this because calculation of the orthogonality score is a computationally burdensome problem. This simplification does not preclude the design of orthogonal pathways and identification of valves in genome-scale networks as the underlying algorithm is based on minimal cut sets that have been extended to genome-scale networks^3^.

**Substrate selection**

The first step in the re-design of metabolic pathways is the selection of a substrate.  Our analysis began by identifying substrates that would make good candidates for biotransformation into our target compound(s).  Any compound that is renewable, inexpensive, non-toxic and capable of being transported into the cytoplasm is a suitable candidate.  Apart from glucose, we found ethylene glycol to satisfy all the above criteria.

**Selection of intermediate precursor(s)**

In the case of orthogonal pathway design, the ability to attain the orthogonal structure in Figure 1a for metabolic pathways is also dependent on the chosen intermediate precursor used as the branching point for metabolic control. Subsequent to identifying a suitable substrate, a suitable precursor metabolite, we need to identify a common metabolite for both product and biomass production pathways. This precursor metabolite should serve as a possible growth substrate for the cell. Pyruvate as an example, is a key precursor from which for instance, succinate can be synthesized. It can also be used as a growth substrate. Since in fact most industrial compounds are produced from a small subset of key metabolic precursors from the central carbon metabolism, the list of suitable precursor metabolites is often very small. Other examples include the production of 1,4-butanediol from acetyl-coA, malonic acid from pyruvate, and isoprenoids from erythrose-4-phospate. These key precursors serve as branch points for building synthetic pathways for natural or non-native compounds. We use a pathway predictor algorithm to identify the synthetic pathways to this precursor metabolite.

**Redox and ATP Cost.**

The conversion of the input substrate must produce reducing equivalents in excess of what is consumed by product formation.  This criterion guarantees that sufficient energy is available for cell growth and maintenance requirements. The criteria arise because energy requirements cannot be met by substrate level phosphorylation.  Since very few enzymes are capable of producing ATP from substrate level phosphorylation, and the metabolites involved in these reactions are also well connected to other parts of the cell’s metabolic network.  This requirement hinders the orthogonality of the network.  Hence, in order to support orthogonal pathways, cellular ATP requirements need to be met by enzymes not involved in substrate level phosphorylation reactions. Oxidative phosphorylation to generate ATP becomes the alternate choice to satisfy cellular ATP needs. Finally, we apply all these criteria in arriving at a suitable synthetic pathway for a given substrate-product pairing.

**Analysis of a Simple Branched Structure**

The main text of the manuscript provides a short analysis of a toy network to provide an understanding of how the orthogonality score is calculated and applied. Here, we provide a short derivation of the theory behind the orthogonality score based on the ideal network structure.

Given an ideal branched structure (Supplementary **Figure 1**), independent from one another and producing either a product P or biomass X, then its elementary flux modes are described by EFM1 and EFM2. This independence arises when no input or co-factor from one branch is required by another. It is readily shown that the orthogonality score for this network is 0.6, as calculated below. Furthermore, in a simple, ideal structure, it can be seen that the score for this network is function of the total length of EFM2 and the substrate utilization reactions, *v_1_* and *v_2_*, up to the branch point metabolite, C. Hence, if there was one more common reaction present between A and C (for a total of 3) then the orthogonality score would decrease to 0.5 or if one less reaction was present between A and C (for a total of 1) then the orthogonality score would increase to 0.75. Thus, this example provides a simplified model of how orthogonality can be calculated and how it is a function of the shared elements (reactions) of the P and X forming EFMs. Hence, in summary, an orthogonality score greater than 0.5 means that fewer than half the reactions are shared relative to the length of the biomass producing EFMs.


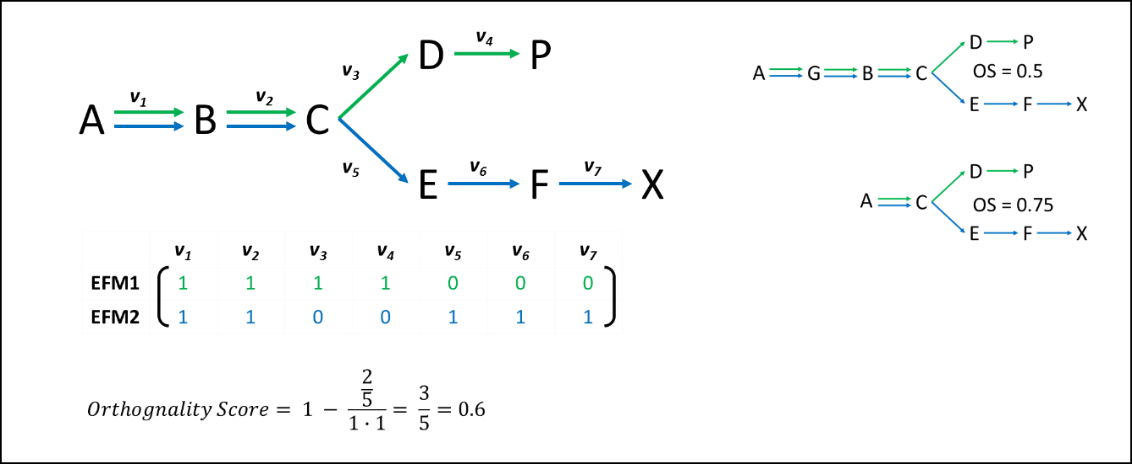


**Supplementary Figure 1.** Orthogonality Calculation for a Branched Structure. EFM1 is indicated in green. EFM2 is indicated in blue.

Let us extend this analysis a little further however to understand how this toy model might apply to a cellular network – and why the orthogonality score as calculated is a suitable metric for larger models.

We begin by noting that that for the branched network structure with two independent arms that there are only two EFMs. The optimal solution has flux through only one branch but the flux distribution, however, can be defined by a linear combination of these two EFMs by

$$\boldsymbol{v}= \sum_{k=1}^{K} \alpha_{k}\boldsymbol{e}_{k}$$

where every flux ***v*** is a non-negative sum of EFMs: $\boldsymbol{e}_{1}\ldots\boldsymbol{e}_{k}$ multiplied by its corresponding weighting factor $\alpha_{k}$ and $\sum\alpha_{k}=1$. In the above example, there are only two EFMs and each is an optimal solution EFM for the production of either P or X. Thus, in the above example, the orthogonality score is determined solely by these two EFMs.

We can also observe that for any network that can produce P or X independently, there will always exist at least two elementary flux modes $\boldsymbol{e}_{k}$ each that is characterized by the smallest set of interactions between the independent production of P and of X. Let these EFMs be $\boldsymbol{e}_{1}$and $\boldsymbol{e}_{2}$ (EFM1 and EFM2 in the above case). Therefore, the largest orthogonality score for the network will exist between these two elementary flux modes and will occur when $\boldsymbol{e}_{1}$ is shortest.

Let us consider that the network is modified in such a way that there is now one additional EFM present in the network, $\boldsymbol{e}_{3}$, such that there are two EFMs producing P and still one producing X. Therefore, the orthogonality score is now also a function of the dot product (shared elements) between $\boldsymbol{e}_{2}$ and $\boldsymbol{e}_{3}$. Since $\boldsymbol{e}_{3}$ is by definition different than $\boldsymbol{e}_{1}$, then by considering this new EFM, the orthogonality score can either increase or decrease. If however, the orthogonality score increases, then this can only occur if the number of reactions shared by $\boldsymbol{e}_{3}$ with $\boldsymbol{e}_{2}$ is less than $\boldsymbol{e}_{1}$ with $\boldsymbol{e}_{2}$. However, if this was the case, then $\boldsymbol{e}_{1}$ is no longer the most orthogonal elementary flux mode as this violates our starting condition. Therefore, $\boldsymbol{e}_{3}$ must have more shared elements with $\boldsymbol{e}_{2}$ than $\boldsymbol{e}_{1}$ has with $\boldsymbol{e}_{2}$ which will necessarily reduce the orthogonality score. It follows, that the addition of any elementary flux mode outside the ideal branched network structure must always reduce the orthogonality score.

Now recognize that the most orthogonal P forming $\boldsymbol{e}_{i}$ to B is not necessarily optimal flux (i.e. highest yield) EFM. Hence, an FBA solution which only finds the optimal flux condition EFM is not sufficient to fully characterize the network interaction space. In natural metabolism, the optimal flux EFM is not necessarily the most orthogonal pathway possible when considering the global repertoire of known metabolic enzymes.

In our present work, we are concerned about the design of substrate utilization pathways and their impact on strain design. In this endeavour, since we are engineering pathways *a priori* we can ignore from consideration those $\boldsymbol{e}_{i}$ that form P but are also low yield since this does not meet design criteria for yield. Hence, it follows that for synthetic pathways like those examples in our case study, or in the simplified branch structure above, the highest yield EFM must also be the most orthogonal. And our central task is to engineer pathways in which the optimal flux EFM corresponds to the most orthogonal EFM from the entire set of possible pathways.

Supplementary Note 2

|  | **Natural Glucose (EMP)** | **Natural Glucose  (ED)** | **Synthetic Glucose** | **Natural**  **Xylose** | **Synthetic Xylose** | **Glycerol** | **Ethylene**  **Glycol 1** | **Ethylene Glycol 2** | **Ethylene Glycol 3** |
| --- | --- | --- | --- | --- | --- | --- | --- | --- | --- |
| Succinic Acid | **0.41**  *82236*  11.2 | **0.45**  *67059*  8.6 | **0.56**  *3610*  3.6 | **0.36**  *86499*  12.8 | **0.57**  *2233*  6.6 | **0.48**  *17943*  9.1 | **0.62**  *464*  3.3 | **0.54**  *1119*  5.2 | **0.36**  *34437* 11.9 |
| Isobutanol | **0.48**  *38202*  10.5 | **0.47**  *29451*  7.7 | **0.54**  *2126*  4.9 | **0.37**  *24798*  12.3 | **0.55**  *2974*  5.9 | **0.49**  *4095*  8.3 | **0.61**  *436*  2.5 | **0.61**  *145*  3.7 | **0.38**  *16663* 11.0 |
| Adipic Acid | **0.44**  *26672*  11.0 | **0.45**  *24114*  8.2 | **0.54**  *1287*  5.5 | **0.35**  *20468*  12.5 | **0.54**  *4025*  5.9 | **0.47**  *4602*  4.7 | **0.57**  *396*  3.3 | **0.52**  *493*  5.2 | **0.34**  *18663* 12.0 |
| Ethanol | **0.44**  *72974*  11.0 | **0.45**  *79170*  8.4 | 0.58  *2319*  4.7 | **0.36**  *67148*  12.4 | **0.54**  *4203*  6.0 | **0.47**  *11510*  8.4 | **0.59**  *1635*  3.8 | **0.61**  263  4.0 | **0.39**  *13967* 11.1 |
| 1,4-Butanediol | **0.46**  *319359*  11*.*7 | **0.44**  *120741*  8.3 | **0.54**  *6356*  6.7 | **0.36**  *198596*  13.3 | **0.55**  *8813*  6.7 | **0.46**  *37430*  9.5 | **0.57**  *1086*  4.2 | **0.53**  *1719* 5.6 | 0.35  *85438* 12.4 |
| 2,3-Butanediol | **0.47**  *24006*  10.5 | **0.48**  *19096*  7.5 | **0.56**  *1756*  3.0 | **0.40**  *16751*  11.9 | **0.55**  *2974*  5.9 | **0.53**  *2652*  7.2 | **0.62**  *436*  2.5 | **0.66**  *91*  3.4 | **0.39**  *25088* 10.7 |

**Supplementary Table 1.  Extended analysis of orthogonality.** The orthogonality scores for a variety of substrates, products and pathways are shown in bold text. The Total Precursor Supporting Reactions are shown in italics while the Average Precursor Reactions/EFM appears as normal text. Broadly, the results support our finding that substrate selection can play in effectively engineering growth independent chemical production. Finally, **Supplementary Figure 2** shows the correlation between the orthogonality scores from **Supplementary Table 1** and the Average Precursor Reactions/EFM that describe the cell’s ability to produce a biomass precursor. The individual pathways used in the modelling along with their metabolic pathways are described in Supplementary Methods.


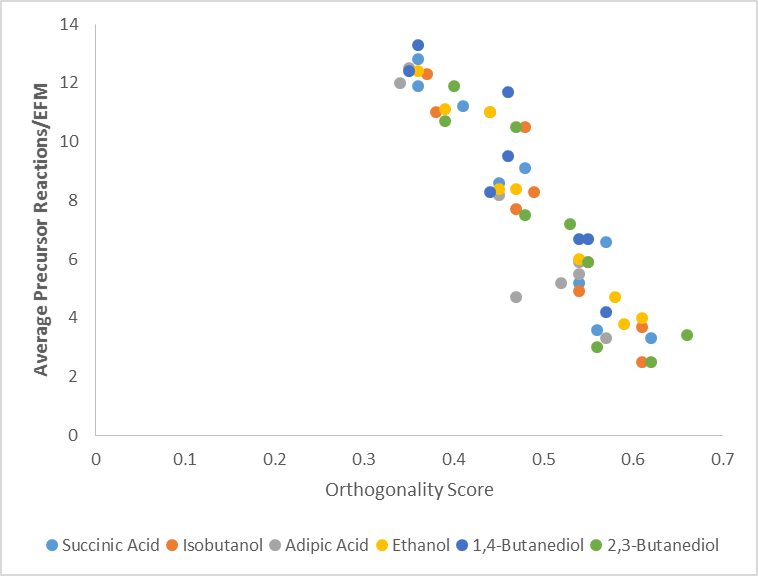


**Supplementary Figure 2. Correlation between Orthogonality Score and the Average Precursor Reactions per EFM.** The following reactions were used to determine the number of biomass precursor forming reactions. {'PGI', 'FBP', 'TKT2', 'TPI', 'FBA', 'GAPD', 'PGK', 'ENO', 'PPCK', 'PPS', 'ME1', 'PYK', 'PDH', 'PPC', 'MDH', 'SUCOAS', 'AKGDH', 'ICDHyr', 'RPI', 'TKT1'};

**Results of orthogonality score and biomass supporting reactions are generalizable**

Production of succinic acid by various different substrates and pathways was used in the main text as a case study to explore concepts of orthogonality and convey the importance that engineering substrate utilization pathways has on various aspects of metabolic engineering. In Supplementary II, our goal is to show that the conclusions derived from the case study is broadly generalizable to various different substrates, their catabolic pathways and across many different products. To that end, we have expanded our analysis to five additional products that have been routinely cited in the literature and are of commercial interest. These products have been explored in four substrates across a total nine different pathways. The orthogonality score for the various combinations of substrates and products are shown above **Supplementary Table 1**. Included in this table is also the total number of elementary flux modes that support biomass pre-cursor forming reactions as well as the average number of biomass pre-cursor forming reactions. Together, these results are meant to mirror the analysis in **Table 1** of the main text. In general, we find consistency between the results in the main text and **Supplementary Table 1**. Below we highlight four observations of interest.

**A Study of Counter Examples**

One benefit of the orthogonality metric for evaluating the predisposition of a metabolic structure to produce a desired chemical is that it reveals, (i) inherent dependency that substrate selection has on growth independent chemical production, and (ii) that the substrate product pairing is not pathway agnostic for ideal networks. These observations, which are made using an unbiased and quantifiable measure, provides a rational basis of design for the metabolic engineer. Two surprising exceptions help to provide a rationale for why several of the observations made in the central text cannot necessarily be derived intuitively for all cases, but rather that a metric provides a basis for understanding how we can achieve orthogonal design of metabolism and rework metabolism in a way that is consequential for metabolic engineering. Hence, first we examine a case where native metabolism is orthogonal towards chemical production and in a second case where a synthetic metabolism is not orthogonal.

**Exception to Natural Metabolism is Not Orthogonal**

We described in the Section 2.2 that natural metabolism was not orthogonal towards succinic acid production. We also described in Section 2.4 of the main text that the substrate product pairing is an essential component of determining how permissible network structures are towards two independent production tasks (biomass and chemical). Those general observations are largely invariant in the presence of the further analysis presented in **Supplementary Table 1**. However, **Supplementary Table 1** does show in interesting result for the production of 2,3-butanediol from glycerol. The orthogonality score for this paring was determined to be 0.53 – greater than any of the natural sugar pathways, though still less than any of the synthetic pathways. Nonetheless a value greater than 0.5 indicates for us a greater ability of the network to support growth independent production. Hence, while instances of natural metabolism exhibiting orthogonal behaviour is uncommon, this exception underscores the role of an unbiased metric rather than intuition in assessing metabolic network structures.

**Exception to Non-native metabolism as obligate orthogonal pathways**

Through the text we describe how synthetic pathways can be used to achieve orthogonal metabolic structures. In the case study on succinic acid, orthogonality was achieved on by way of synthetic pathways but at the same time, it was also dependent on the substrate selection (ex. xylose). Here we provide another exception to the idea that non-native metabolism and pathways are always orthogonal pathways which arises from **Supplementary Table 1** as in the last case, we believe this exception strengthens our case in the text.

The first is presented by examining the three different pathways of ethylene glycol utilization. Despite being a non-native substrate for *E*. *coli*, the degree to which ethylene glycol is capable of supporting biomass independent chemical production is highly dependent on the pathway and the location the pathway enters the cell’s natural metabolism. Ethylene glycol variant #3 has substantially lower scores than variant #2 and #1 because it enters the pentose phosphate pathway. Hence, this finding mirrors our work in the central text that examines xylose utilization through the pentose phosphate pathway or the Weimberg pathway. It supports the idea that not all substrate utilizing pathways evoke a similar behaviour in the cell towards chemical production and that substrate utilization needs to be considered on a case by case basis.

**Co-factor and other network effects are not captured by a simple branched reaction network**

Orthogonality was determined by the elementary flux modes of the network and we found that branched structures exhibited the highest scores. This can lead one, falsely, to the conclusion that it is self-evident that synthetic pathways with a branched topology are orthogonal. To dissuade the reader from making generalizations on the network topology without examining the underlying characteristics of the network, we looked for a counter example that showed a branched structure, but upon determining elementary flux modes and calculating the orthogonality score led one to the opposite conclusion. We find that glycerol conversion to 1,3-propanediol to be an ideal example of the case (**Supplementary** **Figure 3**).

Glycerol can be converted to the product or to biomass by either branch of this ideal network structure. Then one would expect that the orthogonality score for this network to produce 1,3-PDO would be very high and the clear branching should allow the network two function independently and performing either tasks. Instead, what we find is that the orthogonality score for this network is only 0.49. In other words, there is considerable overlap, more than to be expected at least by quick inspection, between the pathways and that this score is far smaller than for networks that appear far more complex (ex. ethylene glycol to succinic acid). Why is this the case? The lower score arises from the network interactions caused by NADH requirements. Thus, this pathway is a simplified example of how co-factor interactions, which are global influencers of the metabolism, might have wide effects that may not be discernable by a cursory look at the pathway. When we take away the NADH requirement for 1,3-PDO synthesis and instead produce 3-hydroxypropionic acid which is more oxidized, the orthogonality score jumps to 0.54.

**
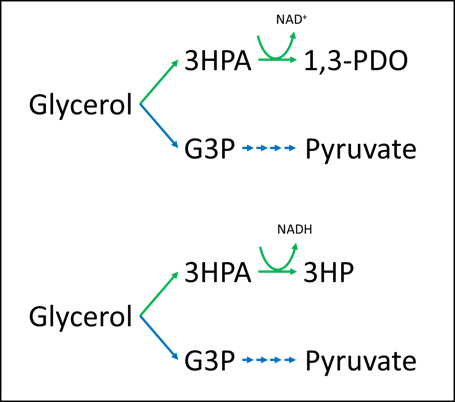
**

**Supplementary Figure 3.** Simplified pathway showing production of 1,3-propanediol (1,3-PDO) and 3-hydroxypropionic acid (3HP) from glycerol. 1,3-PDO requires excess NADH while 3HP does not.

**The Pentose Phosphate Pathway is not suitable for growth and production independence**

From **Supplementary Table 1** we find in general that natural pathways that utilize xylose for the biological production of chemicals are substantially worse in their ability to uncouple product and biomass pathways. These results provide a theoretical basis for understanding xylose or other substrate assimilation pathways that do not pass through the pentose phosphate pathway. The natural xylose pathway and the ethylene glycol variant #3 are both examples of these types of pathways and exhibit the lowest orthogonality scores.

**2.3 Valve Selection is also a determinant of metabolic independence**

A complete list of the cut-sets determined by the ValveFind algorithm for glucose conversion of succinic acid by the synthetic pathway studied in this publication is shown in Supplementary Data. Additionally, **Supplementary Table 2** shows a sample cut-set for the most orthogonal cases of xylose and ethylene glycol utilization for the different product pairing identified in **Supplementary Table 1** as evidence that the approaches and the conclusions laid out in the case study on succinic acid are generalizable to a variety of products, substrates and pathways for substrate assimilation. Out of this analysis, we wanted to highlight a particular finding that sheds novelty in our approach to designing cells with a focus on substrate utilization, namely the selection of reactions suitable as metabolic valves.

Any given cut-set contains a set of genetic deletions as well as the identification of a metabolic valve reaction. In many of these cases, however, more than one reaction is identified as a candidate metabolic valve for a different set of deletions. The question then arises which reaction is more suitable? For example, consider the following designs from Supplementary III.

1. Score: 0.53, Valve: G6PDH2r, Cutset: TKT2 ME1 NADTRHD PPCK
2. Score: 0.51, Valve: TKT2, Cutset: G6PDH2r ME1 NADTRHD PPCK

Despite the similarity in the design, G6PDH2r is suggested to be a slightly better valve that TKT2. Given that recent developments by Prather *et al*. in controlling glucose utilizing reactions^2^ in metabolic engineering, this example is also grounded in physiological reality. The results point to the notion that metabolic control based in rational design may be an essential component of practically realizing these types of dynamic strategies in industrial strains. In another case cut-sets (3) and (4) are reasonably similar in that the deletion set differs by only 1 reaction (PYK vs PPS) yet the metabolic valve selection and the orthogonality score differ substantially supporting the earlier conclusions that network interactions not immediately discernible are systemic and influence strain design.

1. Score: 0.41, Valve: PGK, Cutset: AKGDH ME1 ME2 PYK
2. Score: 0.55, Valve: PPCK, Cutset: AKGDH ME1 ME2 PPS

|  | **Production Envelope, Orthogonality Score and Genetic Strategy** | | |
| --- | --- | --- | --- |
| **Ethylene Glcyol (Pathway I)** | 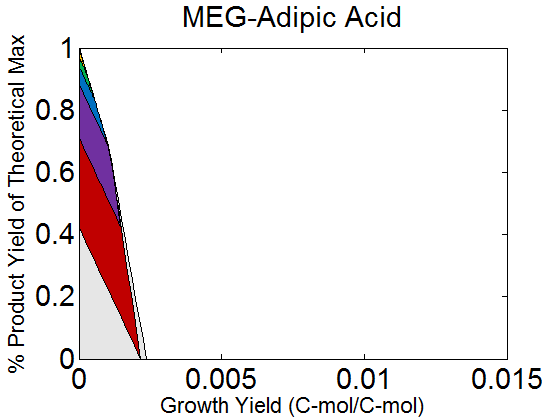  Score: 0.55  Valve: PPCK  Cutset: SUCOAS, PDH, PPS | 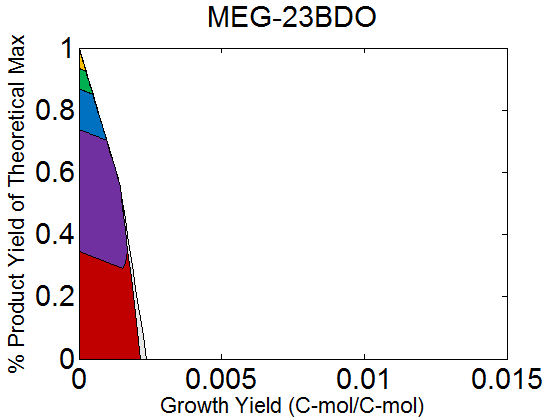  Score: 0.63  Valve: MDH  Cutset: FUM, PDH, PPS | 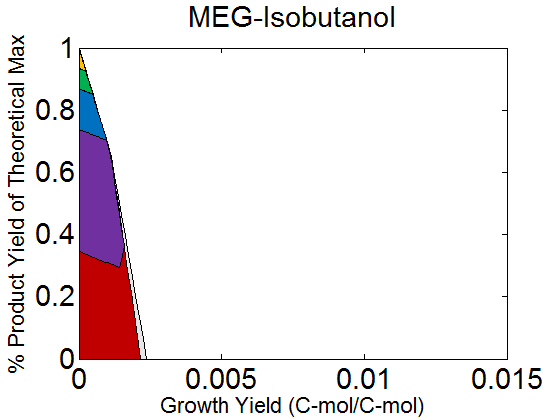  Score: 0.61  Valve: MDH  Cutset: FUM, PDH, PPS |
|  | 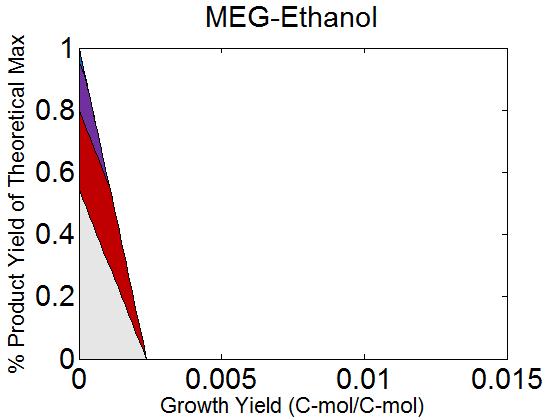  Score: 0.57  Valve: NADH16  Cutset: None |  |  |
| **Synthetic Xylose (Weimberg)** | 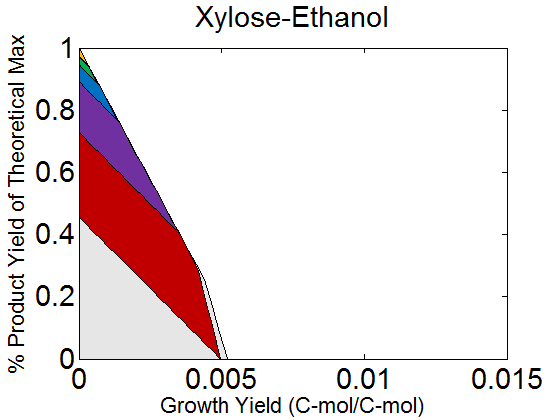  Score: 0.54  Valve: PPS  Cutset: MDH | 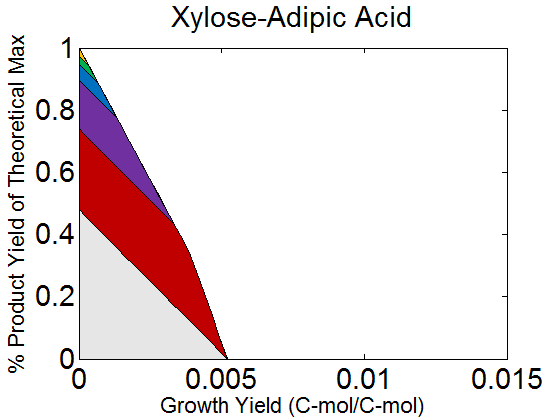  Score: 0.52  Valve: MDH  Cutset: PPS | 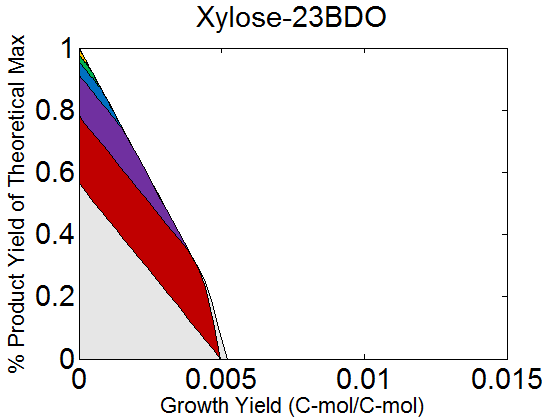  Score: 0.54  Valve: PPS  Cutset: ICDHyr, ICL, PPC |
|  | 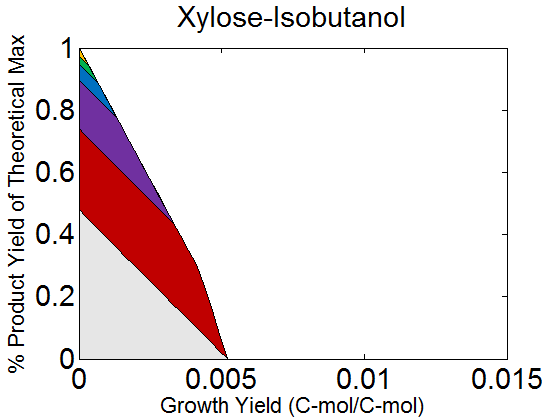  Score: 0.54  Valve: MDH  Cutset: PPS |  |  |

**Supplementary Table 2.  Additional Case Studies on Orthogonal Designs.** The production envelopes and genetic strategies for the most orthogonal pathways are shown as additional validation of the ValveFind methodology.

| **Substrate** | **Cost ($/kg)**  **Cost ($/g Carbon)** | **Pyruvate Yield**  **mol/mol and kg/kg** |
| --- | --- | --- |
| Glucose (as HFCS)^5^ | $0.20-0.66/kg  $0.50-2.45/kg carbon | 2/0.97 |
| Xylose (est. as total cellulosic sugar pricing)^6^ | $0.35/kg  $0.88/kg carbon | 0.6/0.97 |
| Ethylene glycol^7^ | $0.30-0.60/kg  $0.78-1.56/kg carbon | 0.5/0.73 |
| Glycerol (crude)^8^ | $0.06-0.20/kg  $0.19-0.64/kg carbon | 1/0.96 |

**Supplementary Table 3** **Cost of carbon substrates.** The cost of the four substrates examined in this study shows a wide range of cost on a kg of carbon basis. While glycerol appears to be the most economical substrate when the yield for pyruvate is considered, surprisingly ethylene glycol is quite competitive with both glucose and xylose pricing.

**Supplementary Methods**

Modelling Methodology:

The following pathways were added to the *E*. *coli* core model^4^, and simulated in Matlab using the Cobra Toolbox 2.0^1^ and CellNetAnalyzer^3^.

Ethylene Glycol 1

R1 = 'MEG + nad[c] -> glald[c] + nadh[c] + h[c]';

R2 ='glald[c] + nad[c] + h2o[c] -> glyc[c] + nadh[c] + 2 h[c]';

R3 = 'glyc[c] + q8[c] -> glx[c] + q8h2[c]';

R4 = 'MEG -> acald[c] + h2o[c]';

Ethylene Glycol 2

R1 = 'MEG + nad[c] -> glald[c] + nadh[c] + h[c]';

R2 ='glald[c] + nad[c] + h2o[c] -> glyc[c] + nadh[c] + 2 h[c]';

R3 = 'glyc[c] + q8[c] -> glx[c] + q8h2[c]';

R4 = '2 glx[c] + h[c] + nadh[c] + atp[c] -> 2pg[c] + co2[c] + nad[c] + adp[c]';

Ethylene Glycol 3

R1 = 'MEG + nad[c] -> glald[c] + nadh[c] + h[c]';

R2 = '2 glald[c] -> D-threose[c]';

R3 = 'D-threose[c] -> D-erythulose[c]';

R4 = 'D-erythulose[c] + atp[c] -> e4p[c] + adp[c]';

ED Pathway

R1 = 'glc[c] + h2o[c] + q8[c] -> glcn[c] + h[c] + q8h2[c]';

R2 = 'glcn[c] + atp[c] -> 6pgc[c] + adp[c] + h[c]';

R3 = '6pgc[c] -> 2ddg6p[c] + h2o[c]';

R4 = '2ddg6p[c] -> pyr[c] + g3p[c]';

R5 = 'glc-D[e] -> glc[c]';

Synthetic Glucose Pathway

R1 = 'glc-D[e] + h[e] -> glc[c] + h[c]';

R2 = 'glc[c] + h2o[c] + nad[c] -> glcn[c] + h[c] + nadh[c]';

R3 = 'glcn[c] -> 2ddglcn[c] + h2o[c]';

R4 = '2ddglcn[c] -> pyr[c] + glyald[c]';

R5 = 'glyald[c] + nad[c] -> glyc-R[c] + h[c] + nadh[c]';

R6 = 'glyc-R[c] -> pyr[c] + h2o[c]';

Glycerol Pathway

R1 = 'glyc <==>';

R2 = 'atp[c] + glyc[c] -> adp[c] + glyc3p[c] + h[c]';

R3 = 'glyc3p[c] + q8[c] -> dhap[c] + q8h2[c]';

R4 = 'glyc -> glyc[c]';

Xylose Pathway

R1 = 'h[e] + xyl-D[e] -> h[c] + xyl-D[c]';

R2 = 'xyl-D[c] -> xylu-D[c]';

R3 = 'atp[c] + xylu-D[c] -> adp[c] + h[c] + xu5p-D[c]';

Synthetic Xylose Pathway

R1 = 'h[e] + xyl-D[e] -> h[c] + xyl-D[c]';

R2 = 'xyl-D[c] + nad[c] -> xylacn[c] + nadh[c] + h[c]';

R3 = 'xylacn[c] + h2o[c] -> xyln[c] + h[c]';

R4 = 'xyln[c] -> 2kdxyln[c] + h2o[c]';

R5 = '2kdxyln[c] -> akgsad[c] + h2o[c]';

R6 = 'akgsad[c] + h2o[c] + nad[c] -> akg[c] + nadh[c] + 2 h[c]';

1,4-Butanediol

R1 = 'succoa[c] + 4 nadh[c] + atp[c] + 4 h[c] + h2o[c] -> 4 nad[c] + bdo[c] + coa[c] + adp[c] + pi[c]';

2,3-Butanediol

R1 = 'h[c] + 2 pyr[c] -> alac-S[c] + co2[c]';

R2 = 'h[c] + alac-S[c] -> acetoin[c] + co2[c]';

R3 = 'acetoin[c] + nadh[c] + h[c] -> 23bdo[c] + nad[c]';

Adipic Acid

R1 = 'accoa[c] + akg[c] + nadh[c] + h[c] -> coa[c] + co2[c] + nad[c] + adipic[c]';

R2 = 'h[e] + adipic[c] -> h[c] + adipic[e]';

Isobutanol

R1 = '2 pyr[c] + 2 nadh[c] -> ibut[c] + 2 co2[c] + 2 nad[c] + h2o[c]';

**Abbreviations Used in Figure 2 and Figure 4**

glc – glucose; g6p – glucose-6-phosphate; f6p – fructose-6-phosphate; fdp –Fructose 1,6-bisphosphate; g3p -glyceraldehyde 3-phosphate; dhap – dihydroxyacetone phosphate; 13dpg - 1,3-bisphosphoglycerate; 3pg – 3 phosphoglycerate; 2pg – 2phosphoglycerate; pep – phosphoenolpyruvate; pyr- pyruvate; accoa – acetyl-coa; cit – citrate; icit – isocitrate; akg – alpha-ketoglutarate; succoa – succinyl-coa; succ- succinate; fum – fumarate; mal – malate; oaa – oxaloacetate; glcn – gluconate; kdg – 2-keto-3-deoxy-gluconate; glyc – glyceraldehyde; ru5p – ribulose-5-phosphate; x5p – xylulose-5-phosphate; r5p – ribose-5-phophate; s7p - sedoheptulose 7-phosphate; e4p – erythrose-5-phosphate; eg – ethylene glycol; glald – glycolaldehyde; glyclt – glycolate; glx – glyoxylate; acald - acetaldehyde


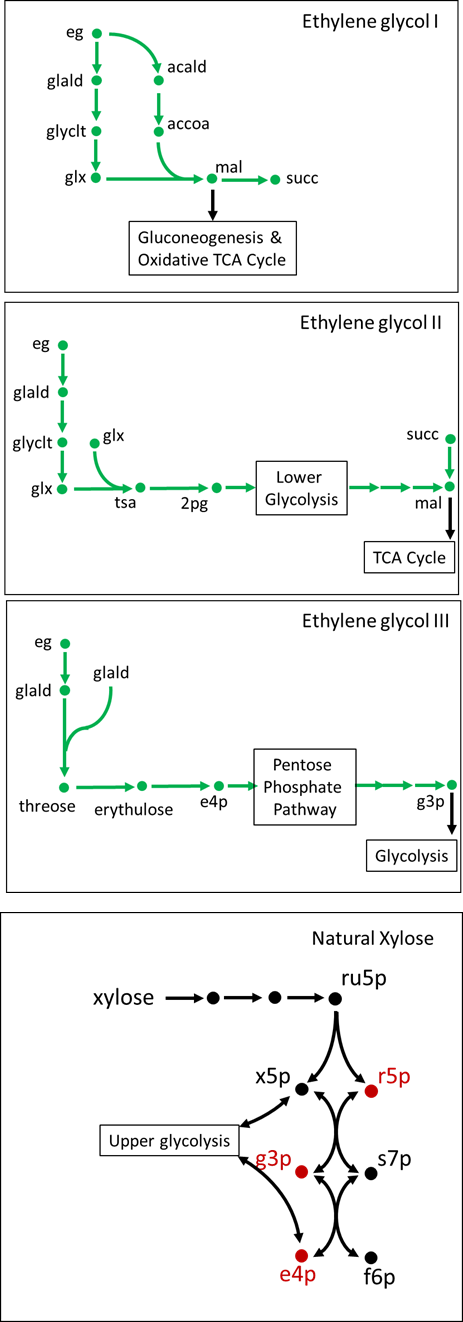


**Supplementary Figure 4.** Substrate utilization pathways examined in this publication are labelled in accordance with **Supplementary** **Table 1**.

**Supplementary References**

1. Hyduke, D. et al., 2011. COBRA Toolbox 2.0. Available at: http://dx.doi.org/10.1038/protex.2011.234.
2. Brockman, I.M. & Prather, K.L.J., 2015. Dynamic knockdown of E. coli central metabolism for redirecting fluxes of primary metabolites. *Metabolic engineering*, 28(5), pp.104–13.
3. Klamt, S. & von Kamp, A., 2011. An application programming interface for CellNetAnalyzer. *Biosystems*, 105(2), pp.162–168. Available at: http://www.sciencedirect.com/science/article/pii/S0303264711000402.
4. Orth J, F.R.P.B., 2010. Reconstruction and Use of Microbial Metabolic Networks: the Core Escherichia coli Metabolic Model as an Educational Guide. *EcoSal Plus*. Available at: http://www.asmscience.org/content/journal/ecosalplus/10.1128/ecosalplus.10.2.1.
5. Table 9--U.S. prices for high fructose corn syrup (HFCS), Midwest markets, monthly, quarterly, and by calendar and fiscal year. USDA (Accessed: 20th December 2016)
6. Lane, J. "Favorable Feedstock Costs Can Drop Cellulosic Sugar Prices As Low As $0.26/Kg : Biofuels Digest". Biofuelsdigest.com. N.p., 2016. (Accessed: 20th December 2016)
7. Raizada, T. US MEG prices likely to rise in January. icis.com Available at: http://www.icis.com/resources/news/2016/12/09/10061977/us-meg-prices-likely-to-rise-in-january/. (Accessed: 20th December 2016)
8. Wong, J. Asia crude glycerine market in stand-off; some sellers keep offers. ICIS News Available at: http://www.icis.com/resources/news/2016/09/08/10032327/asia-crude-glycerine-market-in-stand-off-some-sellers-keep-offers/. (Accessed: 20th December 2016)
